# Midfrontal mechanisms of performance monitoring continuously adapt to incoming information during outcome anticipation

**DOI:** 10.1016/j.ynirp.2023.100182

**Published:** 2023-09-04

**Authors:** Leon Lange, Joanna Kisker, Roman Osinsky

**Affiliations:** a Differential Psychology & Personality Research, Institute of Psychology, Osnabrück University, Germany; b Experimental Psychology I, Institute of Psychology, Osnabrück University, Germany

**Keywords:** Frontomedial theta, Midfrontal theta, FMT, Performance monitoring, VR

## Abstract

Performance monitoring is essential for successful action execution and previous studies have suggested that frontomedial theta (FMT) activity in scalp-recorded EEG reflects need for control signaling in response to negative outcomes. However, these studies have overlooked the fact that anticipating the most probable outcome is often possible. To optimize action execution, it is necessary for the time-critical performance monitoring system to utilize continuously updated information to adjust actions in time. This study used a combination of mobile EEG and virtual reality to investigate how the performance monitoring system adapts to continuously updated information during brief phases of outcome evaluation that follow action execution. In two virtual shooting tasks, participants were either able to observe the projectile and hence anticipate the outcome or not. We found that FMT power increased in response to missing shots in both tasks, but this effect was suppressed when participants were able to anticipate the outcome. Specifically, the suppression was linearly related to the duration of the anticipatory phase. Our results suggest that the performance monitoring system dynamically integrates incoming information to evaluate the most likely outcome of an action as quickly as possible. This dynamic mode of performance monitoring provides significant advantages over idly waiting for an action outcome before getting engaged. Early and adaptive performance monitoring not only helps prevent negative outcomes but also improves overall performance. Our findings highlight the crucial role of dynamic integration of incoming information in the performance monitoring system, providing insights for real-time decision-making and action control.

## Highlights

• The feedback-related FMT response in a virtual shooting task is suppressed if the projectile can be observed.
• As the duration of the anticipatory phase increases, the suppression of feedback-related FMT increases on trial level.
• The performance monitoring system continuously draws on all available information to evaluate an outcome that is likely to occur.

## 1 Introduction

Continuous performance monitoring serves as a fundamental mechanism for the effective regulation of behavior in response to dynamic environmental demands. A crucial aspect of this process is the detection and signaling of increases in need for control triggered by specific events, ranging from cognitive conflicts to behavioral errors and negative performance feedback. Accumulating evidence from recent research has highlighted the presence of a generic need for control signal in the form of transient frontomedial theta activity (FMT) activity (Cavanagh and Frank, 2014; Cavanagh et al., 2012b; M. Cohen, 2016; Watanabe et al., 2021). FMT in the context of performance monitoring is an increased event-related activity in the theta frequency band (4–8 Hz) that shows a frontomedial topography and is suggested to originate in the posterior medial frontal cortex (Cavanagh and Frank, 2014; Cavanagh et al., 2010; Debener et al., 2005; Hanslmayr et al., 2008; Wang, 2005; Yeung et al., 2004).

Increased FMT activity is found in response to conflict inducing stimuli, for instance, in Simon tasks or Flanker tasks (Cavanagh et al., 2012b; M. Cohen and Donner, 2013; M. Cohen and Ridderinkhof, 2013; Duprez et al., 2020; Lange et al., 2022; Pastötter et al., 2013), in response to negative outcomes in gambling tasks (M. Cohen et al., 2007; Gheza et al., 2018; Mueller et al., 2015) as well as to negative feedback in reinforcement learning tasks (Cavanagh et al., 2012a; Cavanagh et al., 2010; Rommerskirchen et al., 2021), where stronger FMT power is associated with enhanced learning effects (van de Vijver et al., 2011). Increased FMT activity in response to behavioral errors has been linked to error awareness (Kalfaoğlu et al., 2018) but has also been shown for implicit errors and, interestingly, does not seem to drive visuomotor adaptation (Jonker et al., 2021). Instead, it is suggested to represent a saliency signal, which conforms to the need for control framework for non-motor errors. It should be noted that there is currently debate about whether FMT is a generic, homogeneous signal (Cavanagh et al., 2012b) or whether there are multiple kinds of different FMTs, possibly with different neural sources, that may be active simultaneously (Lange et al., 2022; Töllner et al., 2017; Zuure et al., 2020). Either way, there appears to be (at least) a general functional role of FMT as a need for control signal (Cooper et al., 2019).

This is also supported by evidence from cued conflict paradigms in which a cue is presented before the actual conflicting stimulus is presented. This cue can contain information whether a conflicting stimulus is about to come up. Generally, the occurrence of conflict increases activity in the anterior cingulate cortex (ACC; Botvinick et al., 2001). However, if a cue indicates that a conflict is about to come up, the ACC exhibits less conflict-related activity when the conflicting stimulus is actually presented (Aarts et al., 2008; Asanowicz et al., 2022; Ide et al., 2013; Luks et al., 2007). In a cued Flanker task, Strack et al. (2013) found that a predictive cue led to attenuation of conflict-related FMT power in response to incongruent trials. Instead, FMT power was increased prior to target onset. In a similar cued task switching paradigm, Cooper et al. (2019) found that switch trials that were preceded by increased FMT power produced smaller switch costs on a behavioral level. Specifically, the performance slowing that is associated with task switching (Jamadar et al., 2015) was attenuated when there was enhanced FMT power while preparing to switch. Increased pre-response FMT activity has been associated with successful trials in different tasks (Cavanagh et al., 2009; Dias et al., 2022; Estiveira et al., 2022; Gomez-Pilar et al., 2018; Ruiz et al., 2011; van Noordt et al., 2017). Thus, early FMT activity seems to help perform the correct action more efficiently. Additionally, the findings on cued conflict paradigms suggest that the need for control signal, indexed by FMT, is triggered by the earliest indicator of an increased need for control, in line increases in FMT power when observing erroneous grasping movements of a virtual agent (Moreau et al., 2021; Pavone et al., 2016).

Here, we extend these findings by specifically investigating the continuous, dynamic adaptations of FMT. Importantly, Cooper et al. (2019) found the association between switch costs and FMT power to be modulated by trial-by-trial changes in theta power. The most common approach to time-frequency analysis on frontomedial theta during cognitive control involves condition-averaged analyses (Cavanagh and Frank, 2014; Cavanagh et al., 2012b; Cooper et al., 2019). However, within-condition averaging eliminates most of its trial-by-trial variance. Therefore, different analyses such as single-trial regressions can provide benefits over cross-trial averaging by considering trial-by-trial variance, in the behavior and in neural dynamics (Cavanagh et al., 2010; M. Cohen and Cavanagh, 2011; Cooper et al., 2019). With this approach, we can explore the potential link between cognitive control and ongoing adaptations.

Single trial regression analyses are well-suited to address two common limitations of previous studies on FMT. First, real-life actions do not always consist of discrete categories like "success" or "failure", "difficult" or "easy", while those are commonly used as laboratory conditions. Instead, many factors naturally vary on a continuous scale. FMT power is sensitive to situational factors such as surprise (Cavanagh et al., 2012a) and punishment expectancy (Cavanagh et al., 2010; Chase et al., 2011; Philiastides et al., 2010). Since these reflect continuous factors, it would be beneficial for experimental settings to implement them as such. Single-trial regression analyses allow for continuous predictors with unique values in every trial instead of discrete conditions.

Second, the FMT-increasing events in laboratory tasks are commonly presented distinctively and at a specific time point. Real life actions, however, often work differently. We are not always dependent on waiting for the completion of the action and feedback on the definitive outcome. Instead, we can monitor many actions permanently and adjust our behavior earlier, if necessary, by anticipating the likely outcome. This allows for corrective action to be taken even before a negative outcome occurs, which is a key aspect of continuous online performance monitoring. Although ERPs provide excellent temporal resolution, they are sensitive to cross-trial latency differences (Luck, 2014). This makes it difficult to map cognitive processes with dynamic timing in traditional ERPs and event-related spectral perturbations (ERSPs). In contrast, regression analyses that consider predictors of just these timing dynamics can simultaneously capture both temporally consistent and temporally varying effects. Thus, applying regression analyses instead of cross-trial averaging opens the possibility to investigate the continuous adaptation of need for control to constantly incoming information.

Therefore, we conducted two experiments to investigate the temporal dynamics of FMT activity. Participants shot at virtual targets in a virtual reality (VR) environment. In the first experiment, they were able to track the trajectory of the projectile, and thus anticipate the outcome before actually hitting the target. In the second experiment, they were not able to track the projectile. We conducted regression analyses for both experiments to estimate how FMT is affected by the outcome (hit/miss), the flight duration of the projectile, and the distance by which the target was missed in the event of a miss. We expect that missing a target increases the FMT power compared to hitting it, as increased FMT power in response to negative outcomes represents a well-established effect (Bernat et al., 2015; Cavanagh et al., 2012a; Cavanagh and Frank, 2014; Cavanagh et al., 2010; M. Cohen, 2016; M. Cohen and Donner, 2013; M. Cohen et al., 2007; Cooper et al., 2019; Fryer et al., 2021; Kalfaoğlu et al., 2018; Lange and Osinsky, 2020; Lange et al., 2022; Philiastides et al., 2010; Töllner et al., 2017; van de Vijver et al., 2011; Wang, 2005; Watanabe et al., 2021; Zuure et al., 2020). Additionally, the regression-based analyses enable individual measures for the flight duration and the distance off target on a continuous scale. The flight duration of the projectiles corresponds to the period during which the participants can observe them and derive anticipations about the most likely outcomes. Interactions of this predictor and outcome-dependent FMT increases, especially in comparison to the experiment without any possibility to observe the projectile, would reflect indicators of dynamic adaptation of need for control to the incoming, observed information. We expect that the ability to observe the trajectory will suppress the outcome-related FMT activity because the outcome can be anticipated in advance, thus reducing the need for control signal that is induced by the actual presentation of the outcome.

## 2 Methods

### 2.1 Participants and procedure

In the first experiment, 23 participants took part. All datasets are included in the final analysis. Thus, data of 23 participants (18 female, 5 male; M_age_ = 22.0 years, SD_age_ = 2.12 years) were analyzed. In the second experiment, 27 participants took part. Three datasets had to be excluded from the analysis due to bad data quality. Thus, data of 24 participants (20 female, 4 male; M_age_ = 22.38 years, SD_age_ = 2.9 years) were analyzed. In both experiments, the sample sizes lead to a power of .96, expecting a strong main effect of the outcome on FMT power (*Cohen's d* = 0.8; J. Cohen, 1988), based on the literature.

All participants had normal or corrected-to-normal vision. Students from the Osnabrück University received course credit for participating in the study. All participants gave written informed consent. The study was approved by the local ethics committee. The procedure in the lab as well as the EEG data recording, processing, and analysis were the same for both experiments.

After arriving at the lab, participants gave written informed consent, filled out a sociodemographic questionnaire, and the EEG was applied. They were equipped with the wireless VR HMD (*HTC Vive Pro*) and VR controllers (*Valve Index*) and performed the experiment task, which took about 20–30 min. The entire procedure took about 60–90 min.

### 2.2 Experimental task design

The virtual environment was created using Unity 5 (Unity Technologies, San Francisco, United States). Within the virtual environment, the subjects saw a forest clearing and picked up a virtual pistol. In each trial, a balloon appeared in a random position and slowly floated upwards. The participants were instructed to shoot this balloon. They had one shot for each balloon/trial. If they hit the balloon, it burst into green fragments, indicating a successful trial. If they missed the balloon, it also burst, but into red fragments, indicating an erroneous trial. Depending on the outcome, the balloon in the next trial spawned closer to the participant after an erroneous trial or farther away after a successful trial (M_distance_ = 35.46m, SD_distance_ = 13.77m). This way, a hit rate of M = 52.32% (SD = 11.23%) was achieved. All participants completed 5 blocks with 100 trials each.

In the first experiment the pistol shot projectiles that could be seen and traced. The projectiles moved at a speed of 195 $\frac{m}{s}$ (M_air time_ = 181.34ms, SD_air time_ = 45.08ms) according to a trajectory affected by gravity. To ensure similar timing of successful and erroneous trials, the exact time point of the balloons bursting was determined by the distance between the participant and the balloon. The balloons burst as soon as the projectile reached the distance between the participant and the balloon. For successful trials this corresponds to the moment when the projectile hits the balloon. In the case of erroneous trials, this means that the balloons burst at the exact moment when the projectile would have hit them if it had not deviated from the target.

The experimental task design in the second experiment was similar to the first experiment with the exception that the virtual pistol shot laser rays instead of projectile. Each ray appeared immediately upon pulling the trigger on the controller and was visible for 100ms. In contrast to the first experiment, the ray hit the balloon instantly and thus the outcome was identifiable immediately. There was no anticipatory phase between pulling the trigger and the bursting of the balloon. Both events happened at the same time, independent of the shooting distance. Similar to the first experiment the distance was adjusted on a trial-by-trial base (M_distance_ = 31.23m, SD_distance_ = 12.13m) and the balloons burst after every shot.

It is important to note that in the first experiment the distance to the target corresponds to the time in which the projectile can be observed. A greater distance leads to a longer observation time and vice versa. In the second experiment, the same factor reflects only the distance, since there is no observation time. Therefore, we will refer to this factor as distance/time in the following.

### 2.3 EEG data recording and processing

EEG data was recorded with a mobile 32 channel active EEG system (Live Amp, Brain Products, Gilching, Germany) using the Brain Vision Recorder software (Brain Products, Gilching, Germany) and synchronized with Unity via Lab Streaming Layer (LSL). The electrode layout was according to the international 10-20-system. The data were recorded with a 500 Hz sampling rate and 0.016–250 Hz band-pass filter. The data was referenced online to FCz and the ground electrode was placed at AFz. The impedance of all electrodes was kept below 15 kΩ.

Offline preprocessing and analysis was conducted with Matlab (MathWorks Inc.) and EEGLAB (Delorme and Makeig, 2004). Line noise was removed using the zapline plus plugin (Klug and Kloosterman, 2022) and channels that were repeatedly detected as bad channels in more than 50% of 20 iterations were removed and interpolated (Klug et al., 2022). Applying this procedure, a total of 10 channels was interpolated in six datasets (M = 0.21, SD = 0.59; Max = 2). The data were further referenced to the common average, the former reference channel FCz was reinstated as additional data channel and the data were filtered with a 1–30 Hz band-pass filter (Klug and Gramann, 2021). Then artifact subspace reconstruction (ASR) was applied for burst correction followed by AMICA (Palmer et al., 2012) as this provides a powerful combination to extract independent components in mobile EEG setups (Chang et al., 2018; Gorjan et al., 2022). The extracted components were classified using the ICLabel plugin and components with a probability >80% of being a muscle or eye component were removed (M = 2.06, SD = 0.84, Max = 5).

**Fig. 1:**
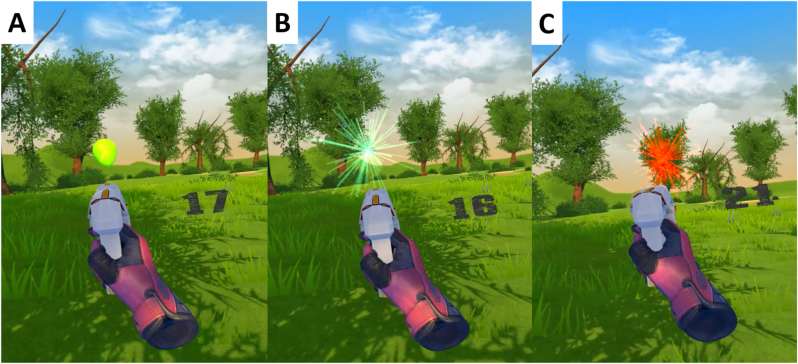
Screenshots of the virtual environment. A: Balloon before shot. B: Green fragments after hitting the balloon. C: Red fragments after missing the balloon. The number to the right displays the number of trials remaining in the given block. (For interpretation of the references to colour in this figure legend, the reader is referred to the Web version of this article.)

Continuous EEG was segmented from −1500ms to 1000ms around the outcome feedback onset, i.e., the burst of the balloon. A time-frequency transformation was applied on the single trial level using a family of complex Morlet wavelets in 30 logarithmic steps. Importantly, for analyzing the data we implemented a relative baseline division per frequency layer (−1000ms to −500ms) without a dB-transformation. We applied dB-transformation only for computing the grand averages of power values that are displayed in Fig. 1, since logarithmic scaling on single trial level would be biased for small values (M. Cohen, 2014).

For the data of the first experiment, we also applied a second analysis where we segmented the continuous EEG around the time point of *pulling the trigger* of the gun (−1500ms–1000ms), i.e., the moment of shooting. All subsequent analysis steps were applied to these segments in the same way. Thus, we analyzed three experimental conditions in total: (1) data from the first experiment (projectile) relative to the moment of the outcome presentation (“*exp. 1 (projectile): outcome”*); (2) data from the first experiment (projectile) relative to the moment of the shot (“*exp. 1 (projectile): shot”*); (3) data from the second experiment (laser), where the moment of the outcome presentation matches the moment of the shot (“*exp. 2 (laser)”*).

### 2.4 Regression analysis

In order to investigate the impact of different continuous factors on single trial level, we computed regression-based ERSPs (rERSPs) (Smith and Kutas, 2015). Using mass-univariate multiple regression, a linear model was estimated according to the following formula:

$$y_{i}=\mathrm{INT}+{\beta_{1}x}_{1i}+\ldots +{\beta_{5}x}_{5i}+{noise}_{i}$$

$$With\;y_{i}=power\;at\;time-frequency\;point\;for\;trial\;i$$

INT=Intercept

$$x_{1}=1\;for\;successful\;trials,0\;for\;erroneous\;trials$$

$$x_{2}=distance\;to\;target\;\left(≙observation\;time\right)$$

$$x_{3}=outcome*\;distance\;to\;target\;\left(≙observation\;time\right)$$

$$x_{4}=error\;size$$

$$x_{5}=error\;size*\;distance\;to\;target\;\left(≙observation\;time\right)$$

The variables for error size and distance/time were z-transformed. The model was estimated for each sample point for each frequency. This derives five regression weights and an intercept for each sample point and frequency per channel and participant. We computed the coefficients for the effects that were not directly resembled by a regressor, leading to a total of eight coefficients that we analyzed (Outcome_Miss_, Outcome_Hit_, Outcome_Miss vs. Hit_, Distance/Time_Miss_, Distance/Time_Hit_, Distance/Time_Miss vs. Hit_, Error Size_Miss_, Interaction: Distance/Time_Miss_*Error Size_Miss_).

We averaged the theta band (4–8 Hz) estimates for each coefficient and applied cluster-based permutation tests to derive time windows with significant effects per coefficient. We used data from channel FCz (based on the literature), cluster-forming thresholds of *p* = .01 and computed 10.000 iterations. We applied the cluster-based permutation tests to the data of each experimental condition, namely e*xp. 1 (projectile): outcome*, *exp. 1 (projectile): shot*, and *exp. 2 (laser)*. In addition, we computed difference scores by subtracting the regression coefficients from *exp. 2 (laser)* from those of *exp. 1 (projectile): shot* and subjected these difference scores to the same cluster-based permutation tests. We excluded the data from *exp. 1 (projectile): outcome* from this analysis, as visual inspection indicated that the timing of the effects was too dissimilar to enable a meaningful comparison.

## 3 Results

**Fig. 2:**
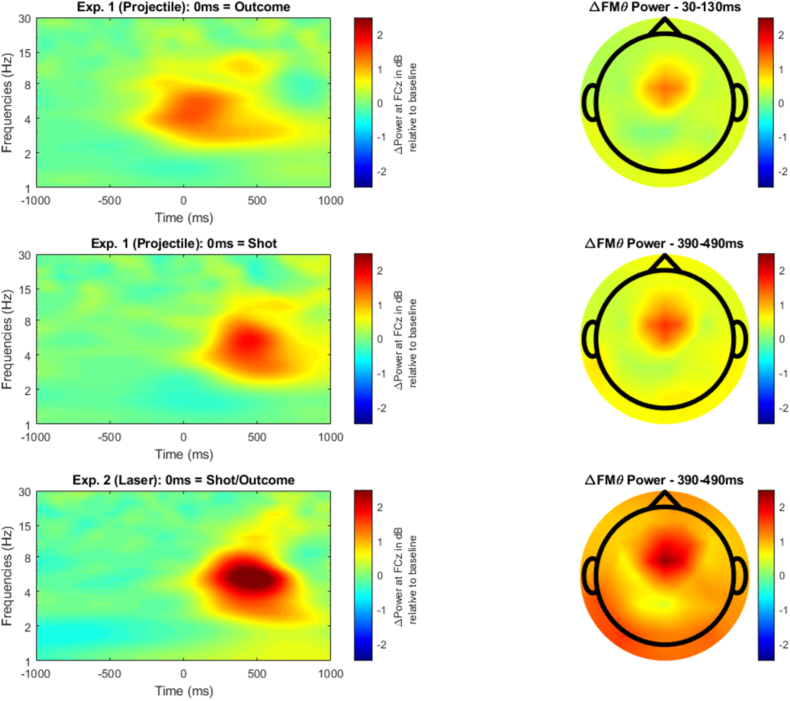
Time-frequency activity for the differences of miss minus hit trials per experimental condition. Time-frequency plots display dB-transformed Δpower at FCz.

**Fig. 3:**
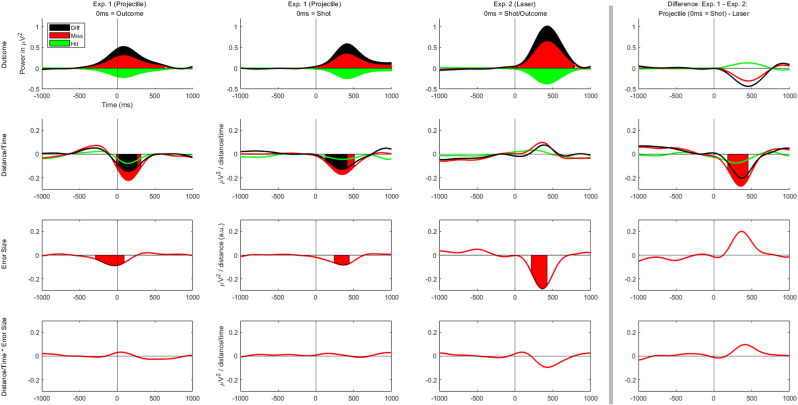
Regression-based ERSPs (rERSPs) for the theta-band (4 Hz–8 Hz) at channel FCz. The ERSPs are based on non dB-transformed theta power. Shaded areas reflect time windows with statistically significant effects of the corresponding regression coefficient on theta power (p < .01).

The dB-transformed power values and the topographies for the differences of miss minus hit trials are displayed in Fig. 2. The results of the regression analyses are displayed in Fig. 3. For all three experimental conditions (exp. 1 (projectile): outcome; exp. 1 (projectile): shot; exp. 2 (laser)) we found a general FMT increase in miss trials (*p* < .00001 [−356ms, 612ms]; *p* < .00001, [14ms, 1000ms]; *p* < .00001, [32ms, 770ms]) and a general FMT decrease in hit trials (*p* < .00001, [−454ms, 620ms]; *p* < .00001, [4ms, 1000ms]; *p* < .00001, [−56ms, 816ms]), meaning that FMT power differs significantly from the baseline. Further, there was significantly stronger FMT power after miss trials compared to hit trials (*p* < .00001, [−408ms, 620ms]; *p* < .0001, [2ms, 1000ms]; *p* < .00001 [−42ms, 786ms]).

Interestingly, the cluster-based permutation tests revealed no significant effects of distance/time for the second experiment (laser) but for the first experiment (projectile; exp. 1 (projectile): outcome; exp. 1 (projectile): shot). For miss trials, there is significantly less FMT power with increasing distance/time (*p* < .00001, [−14ms, 314ms]; *p* < .00001, [120ms, 508ms]) when a traceable projectile was visible. This effect is descriptively smaller but also significant for hit trials segmented relative to the outcome (*p* = .002, [16ms 250ms]) but is not apparent for hit trials segmented relative to the shot. For both segmentations, the distance/time effect for miss trials is significantly stronger than the distance/time effect for hit trials (*p* < .00001, [20ms, 264ms]; *p* = .001, [140ms, 412ms]), but again, this is not the case for exp. 2 (laser).

We found decreasing FMT power with increasing error size in miss trials for all three experimental conditions again (*p* < .00001, [−288ms, 92ms]; *p* = .003, [242ms, 440ms]; *p* < .00001, [216ms, 424ms]). There were no significant interactions of distance/time and error size.

Finally, we compared the two experiments using the regression coefficients of exp. 1 (projectile): shot and exp. 2 (laser). The cluster-based permutation tests revealed only one significant cluster, namely the effect of less FMT power with increasing distance/time for miss trials was stronger in the first experiment (projectile) than in the second experiment (laser; *p* = .002, [180ms, 450ms]).

## 4 Discussion

We investigated the integration of continuously incoming information in the performance monitoring system using two shooting tasks in immersive virtual environments. In one task, it was possible to observe the trajectory of the projectile and in the other it was not. Our results show that the observation of the trajectory influences the cognitive processing of the outcome. The performance monitoring system seems to continuously draw on all available information to evaluate as quickly as possible an outcome that is likely to occur. The FMT response to the definite outcome feedback is attenuated if the trajectory of the projectile can be observed beforehand. This is in line with the framework that FMT acts as a need for control signal and is therefore not needed when the negative outcome is already expected due to the observation of the trajectory.

As expected, we found increased FMT after missing shots in both experiments. This finding corresponds to well-established effects of increased FMT in response to errors or negative feedback (Cavanagh et al., 2012a; M. Cohen et al., 2007; Gheza et al., 2018; Jonker et al., 2021; Kalfaoğlu et al., 2018; Mueller et al., 2015; van de Vijver et al., 2011). However, in our experiments, FMT power was also impacted by the ability to observe the projectile. When the participants were able to observe the projectile, the FMT power decreased linearly with increasing distance to the target/observation time. Crucially, we did not find any effect of distance on FMT power when the participants were not able to observe the projectile. Instead, distance had a significantly stronger FMT decreasing effect when participants could observe the projectile, than when they could not. Thus, the distance to a target itself does not seem to evoke this suppression in FMT. Rather, the observation time seems to drive this effect, since it corresponds to the distance in the experiment where participants can observe the projectile but is missing in the other experiment. Accordingly, the longer participants can observe the projectile, the more the outcome-related FMT is attenuated. This effect is similar to the results of cued conflict paradigms on ACC activity in the fMRI (Aarts et al., 2008; Asanowicz et al., 2022; Ide et al., 2013; Luks et al., 2007) and on FMT in the EEG (Cooper et al., 2019; Strack et al., 2013). It supports the assumption that FMT reflects a signal of the performance monitoring system to indicate a need for control. A sudden negative event elicits a high need for control when no information is available before an outcome is presented. However, if incoming information about a likely outcome is available, it seems to be continuously integrated by the performance monitoring system. By such continuous evidence-accumulation, the performance monitoring system may already “know” about the outcome at the moment it is presented. Thereby the sudden increase in need for control at that single moment is dampened because adaptations can be prepared and initiated in advance.

Such a dynamic, continuous mode of performance monitoring would provide obvious advantages for the organism, as opposed to, in the most extreme case, waiting for an action to end before evaluating its outcome. In this framework, the anticipatory online evaluation should draw on multidimensional sources of information to achieve high degrees of precision and especially expectations, which are derived, updated, and integrated on an ongoing basis. One could define several types of expectations, possibly even infinitely many with continuous transitions between them. Following, we will differentiate three main categories: global, intermediate, and local expectations.

When performing a task repeatedly, either one time right after the other or because it is a mundane task that we might have done many times before, we generate a global expectancy about the probability of every possible outcome. Expectations are further generated within a smaller, intermediate framework. Research on different ERP components has shown that performance monitoring in a given trial is influenced by the outcome of the immediately preceding trials or events. One example of a prominent behavioral effect found across tasks is post-error slowing/speeding (Damaso et al., 2020; Danielmeier and Ullsperger, 2011; Dudschig and Jentzsch, 2009; Purcell and Kiani, 2016), but also at the neuronal level, preceding events can cause an amplification/decrease in the effects of feedback-related activity (Osinsky et al., 2012) and FMT power (Pastötter et al., 2013) in subsequent trials. The history of preceding events is integrated to build up sequence-generated expectancies, which are less stable than the global expectations and must be dynamically adjusted after each new event. Previous studies have shown that the probability of a specific outcome affects the outcome-related FMT power, with increased power for unexpected outcomes (Cavanagh et al., 2012a; Cavanagh et al., 2010; Gheza et al., 2019; Hajihosseini and Holroyd, 2013; Janssen et al., 2016; Osinsky et al., 2016; Paul et al., 2020; Rommerskirchen et al., 2021; Umemoto et al., 2023). While this is in line with the framework of FMT as a need for control signal, these experiments only took the global and intermediate expectations into account, since the probability manipulations were implemented trial or condition wise with high vs. low outcome probabilities or expected vs. unexpected outcomes. Our study revealed that outcome evaluations in the medial frontal cortex, indicated by FMT (Cavanagh and Frank, 2014; Cavanagh et al., 2010; Debener et al., 2005; Hanslmayr et al., 2008; Wang, 2005; Yeung et al., 2004), are not only sensitive to global and intermediate expectations but also to expectations at an even smaller, local level. These local expectations are built, evaluated, and discarded within a single trial while observing the projectile. The expectation formed before the trial can be continuously updated based on the constant flow of new information about the location and trajectory of the projectile. This enables the anticipation of the outcome to be improved continuously, and not exclusively depend on pre-trial expectations that are based on global and intermediate contexts. The continuous online integration of information allows for the control signal to be adjusted, even before the definite outcome occurs. This explains, on the one hand, why informative cues about a pending conflict suppress ACC activity (Aarts et al., 2008; Asanowicz et al., 2022; Ide et al., 2013; Luks et al., 2007) and FMT (Cooper et al., 2019; Strack et al., 2013). On the other hand, it also explains the linear relationship between observation duration and FMT suppression found in our study. The longer the projectile can be observed, the more local information is available in the online evaluation, leading to improved anticipation accuracy and corresponding adjustments in the need for control signal.

Apart from the effect of target distance on FMT, we also observed a negative linear relationship between error size and FMT power in both shooting tasks. Apparently, this contrasts with some previous studies that found larger errors during continuous movement tasks to elicit an enhanced increase in FMT power (Arrighi et al., 2016; Jonker et al., 2021; Spinelli et al., 2018) or the functionally related error-related negativity (ERN; Vocat et al., 2011). In our view, the negative relationship observed in our study reflects the effects of anticipation and expectancy in our experimental design. When participants were able to observe the projectile, larger errors may have been easier to detect with greater confidence during the anticipatory phase. This could have provided a larger and stronger base of evidence for performance monitoring, enabling the anticipation of the outcome and attenuating the feedback-related FMT activity. Interestingly, we also observed a negative relationship between FMT power and error size in the second experiment where the projectile could not be observed. One potential explanation is that this reflects an effect of expectancies which are built during the phase of aiming and taking the shot. Poorly executed actions might already be associated with a negative outcome expectation, resulting in a weaker increase in FMT power. In our experiment, corrective actions could not be taken during the trial, which may have contributed to the weaker association between FMT power and error size.

Furthermore, continuous adjustment of expectations may affect performance adjustments during the anticipation phase. In many daily actions, it is appropriate or even existentially necessary that the performance monitoring system not idly waits for the outcome before intervening, e.g., when we misjudge the speed while taking a turn in a car or when we reach for a glass of water but miss it by a few inches and knock it over. Rather, the performance monitoring system should continuously collect and evaluate information about the likely outcome of the action before it occurs to use this information for proactive control and adaptive processes of executive control already during action performance (Alexander and Brown, 2011; Braver et al., 2009). Another limitation to be addressed is the unbalanced gender distribution in our samples, which could have affected the results. While these are only post hoc explanations which call for future verification our findings highlight the importance of an accurate differentiation of task characteristics, as the interplay of task demands, cognitive processes, and individual differences seems to contribute to a complex variability in the relationship between FMT power and error size across different contexts (Pastötter et al., 2012).

Our experiment has shown that the performance monitoring system is continuously drawing on all available information to evaluate an outcome that is likely to occur and adapting its need for control signal accordingly. This dynamic mode of operation can significantly improve performance monitoring. Fast and accurate online evaluations of actions are critical in everyday life in order to be able to adjust behavior in time. Accordingly, it seems only reasonable that our performance monitoring system is optimized to not only effectively learn from mistakes, but also to avoid them, if possible, before or even while they are being made.

## Author contributions

**Leon Lange**: Conceptualization; Investigation; Formal analysis; Writing - original draft, review & editing. **Joanna Kisker**: Conceptualization; Investigation; Writing - review & editing. **Roman Osinsky**: Conceptualization; Supervision; Writing - review & editing.

## Declaration of competing interest

The authors declare that they have no known competing financial interests or personal relationships that could have appeared to influence the work reported in this paper.

## Data Availability

All data of the present study will be made fully available on OSF (https://osf.io) prior to a potential publication in NeuroImage: Reports.

## References

[bib1] Aarts E., Roelofs A., van Turennout M. (2008). Anticipatory activity in anterior cingulate cortex can be independent of conflict and error likelihood. J. Neurosci..

[bib2] Alexander W.H., Brown J.W. (2011). Medial prefrontal cortex as an action-outcome predictor. Nat. Neurosci..

[bib3] Arrighi P., Bonfiglio L., Minichilli F., Cantore N., Carboncini M.C., Piccotti E., Rossi B., Andre P. (2016). EEG theta dynamics within frontal and parietal cortices for error processing during reaching movements in a prism adaptation study altering visuo-motor predictive planning. PLoS One.

[bib4] Asanowicz D., Kotlewska I., Panek B. (2022). Neural underpinnings of proactive and preemptive adjustments of action control. J. Cognit. Neurosci..

[bib5] Bernat E.M., Nelson L.D., Baskin-Sommers A.R. (2015). Time-frequency theta and delta measures index separable components of feedback processing in a gambling task. Psychophysiology.

[bib6] Botvinick M.M., Braver T.S., Barch D.M., Carter C.S., Cohen J.D. (2001). Conflict monitoring and cognitive control. Psychol. Rev..

[bib7] Braver T.S., Paxton J.L., Locke H.S., Barch D.M. (2009).

[bib8] Cavanagh J., Cohen M., Allen J.J.B. (2009). Prelude to and resolution of an error: eeg phase synchrony reveals cognitive control dynamics during action monitoring. J. Neurosci..

[bib9] Cavanagh J., Figueroa C.M., Cohen M., Frank M.J. (2012). Frontal theta reflects uncertainty and unexpectedness during exploration and exploitation. Cerebral Cortex (New York, N.Y. : 1991.

[bib10] Cavanagh J., Frank M. (2014). Frontal theta as a mechanism for cognitive control. Trends Cognit. Sci..

[bib11] Cavanagh J., Frank M., Klein T., Allen J.J.B. (2010). Frontal theta links prediction errors to behavioral adaptation in reinforcement learning. Neuroimage.

[bib12] Cavanagh J., Zambrano-Vazquez L., Allen J.J.B. (2012). Theta lingua franca: a common mid-frontal substrate for action monitoring processes. Psychophysiology.

[bib13] Chang C.-Y., Hsu S.-H., Pion-Tonachini L., Jung T.-P. (2018). Annual International Conference of the IEEE Engineering in Medicine and Biology Society. IEEE Engineering in Medicine and Biology Society. Annual International Conference, 2018.

[bib14] Chase H.W., Swainson R., Durham L., Benham L., Cools R. (2011). Feedback-related negativity codes prediction error but not behavioral adjustment during probabilistic reversal learning. J. Cognit. Neurosci..

[bib15] Cohen J. (1988).

[bib16] Cohen M. (2014).

[bib17] Cohen M. (2016). Midfrontal theta tracks action monitoring over multiple interactive time scales. Neuroimage.

[bib18] Cohen M., Cavanagh J. (2011). Single-trial regression elucidates the role of prefrontal theta oscillations in response conflict. Front. Psychol..

[bib19] Cohen M., Donner T.H. (2013). Midfrontal conflict-related theta-band power reflects neural oscillations that predict behavior. J. Neurophysiol..

[bib20] Cohen M., Elger C.E., Ranganath C. (2007). Reward expectation modulates feedback-related negativity and EEG spectra. Neuroimage.

[bib21] Cohen M., Ridderinkhof K.R. (2013). Eeg source reconstruction reveals frontal-parietal dynamics of spatial conflict processing. PLoS One.

[bib22] Cooper P.S., Karayanidis F., McKewen M., McLellan-Hall S., Wong A.S.W., Skippen P., Cavanagh J.F. (2019). Frontal theta predicts specific cognitive control-induced behavioural changes beyond general reaction time slowing. Neuroimage.

[bib23] Damaso K., Williams P., Heathcote A. (2020). Evidence for different types of errors being associated with different types of post-error changes. Psychonomic Bull. Rev..

[bib24] Danielmeier C., Ullsperger M. (2011). Post-error adjustments. Front. Psychol..

[bib25] Debener S., Ullsperger M., Siegel M., Fiehler K., Cramon D. Y. von, Engel A.K. (2005). Trial-by-trial coupling of concurrent electroencephalogram and functional magnetic resonance imaging identifies the dynamics of performance monitoring. J. Neurosci..

[bib26] Delorme A., Makeig S. (2004). Eeglab: an open source toolbox for analysis of single-trial EEG dynamics including independent component analysis. J. Neurosci. Methods.

[bib27] Dias C., Costa D., Sousa T., Castelhano J., Figueiredo V., Pereira A.C., Castelo-Branco M. (2022). A neuronal theta band signature of error monitoring during integration of facial expression cues. PeerJ.

[bib28] Dudschig C., Jentzsch I. (2009). Speeding before and slowing after errors: is it all just strategy?. Brain Res..

[bib29] Duprez J., Gulbinaite R., Cohen M.X. (2020). Midfrontal theta phase coordinates behaviorally relevant brain computations during cognitive control. Neuroimage.

[bib30] Estiveira J., Dias C., Costa D., Castelhano J., Castelo-Branco M., Sousa T. (2022). An action-independent role for midfrontal theta activity prior to error commission. Front. Hum. Neurosci..

[bib31] Fryer S.L., Roach B.J., Holroyd C.B., Paulus M.P., Sargent K., Boos A., Ford J.M., Mathalon D.H. (2021). Electrophysiological investigation of reward anticipation and outcome evaluation during slot machine play. Neuroimage.

[bib32] Gheza D., Bakic J., Baeken C., Raedt R. de, Pourtois G. (2019). Abnormal approach-related motivation but spared reinforcement learning in MDD: evidence from fronto-midline Theta oscillations and frontal Alpha asymmetry. Cognit. Affect Behav. Neurosci..

[bib33] Gheza D., Raedt R. de, Baeken C., Pourtois G. (2018). Integration of reward with cost anticipation during performance monitoring revealed by ERPs and EEG spectral perturbations. Neuroimage.

[bib34] Gomez-Pilar J., Poza J., Gómez C., Northoff G., Lubeiro A., Cea-Cañas B.B., Molina V., Hornero R. (2018). Altered predictive capability of the brain network EEG model in schizophrenia during cognition. Schizophr. Res..

[bib35] Gorjan D., Gramann K., De Pauw K., Marusic U. (2022). Removal of movement-induced EEG artifacts: current state of the art and guidelines. J. Neural. Eng..

[bib36] Hajihosseini A., Holroyd C.B. (2013). Frontal midline theta and N200 amplitude reflect complementary information about expectancy and outcome evaluation. Psychophysiology.

[bib37] Hanslmayr S., Pastötter B., Bäuml K.-H., Gruber S., Wimber M., Klimesch W. (2008). The electrophysiological dynamics of interference during the Stroop task. J. Cognit. Neurosci..

[bib38] Ide J.S., Shenoy P., Yu A.J., Li C.R. (2013). Bayesian prediction and evaluation in the anterior cingulate cortex. J. Neurosci..

[bib39] Jamadar S.D., Thienel R., Karayanidis F. (2015). Task switching processes. Brain Mapping.

[bib40] Janssen D.J.C., Poljac E., Bekkering H. (2016). Binary sensitivity of theta activity for gain and loss when monitoring parametric prediction errors. Soc. Cognit. Affect Neurosci..

[bib41] Jonker Z.D., van der Vliet R., Maquelin G., van der Cruijsen J., Ribbers G.M., Selles R.W., Donchin O., Frens M.A. (2021). Individual differences in error-related frontal midline theta activity during visuomotor adaptation. Neuroimage.

[bib42] Kalfaoğlu Ç., Stafford T., Milne E. (2018). Frontal theta band oscillations predict error correction and posterior slowing in typing. J. Exp. Psychol. Hum. Percept. Perform..

[bib43] Klug M., Gramann K. (2021). Identifying key factors for improving ICA-based decomposition of EEG data in mobile and stationary experiments. Eur. J. Neurosci..

[bib44] Klug M., Jeung S., Wunderlich A., Gehrke L., Protzak J., Djebbara Z., Argubi-Wollesen A., Wollesen B., Gramann K. (2022).

[bib45] Klug M., Kloosterman N.A. (2022). Zapline-plus: a Zapline extension for automatic and adaptive removal of frequency-specific noise artifacts in M/EEG. Hum. Brain Mapp..

[bib46] Lange L., Osinsky R. (2020). Aiming at ecological validity-Midfrontal theta oscillations in a toy gun shooting task. Eur. J. Neurosci..

[bib47] Lange L., Rommerskirchen L., Osinsky R. (2022). Midfrontal theta activity is sensitive to approach-avoidance conflict. J. Neurosci..

[bib48] Luck S.J. (2014).

[bib49] Luks T.L., Simpson G.V., Dale C.L., Hough M.G. (2007). Preparatory allocation of attention and adjustments in conflict processing. Neuroimage.

[bib50] Moreau Q., Tieri G., Era V., Aglioti S.M., Candidi M. (2021).

[bib51] Mueller E.M., Panitz C., Pizzagalli D.A., Hermann C., Wacker J. (2015). Midline theta dissociates agentic extraversion and anhedonic depression. Pers. Indiv. Differ..

[bib52] Osinsky R., Mussel P., Hewig J. (2012). Feedback-related potentials are sensitive to sequential order of decision outcomes in a gambling task. Psychophysiology.

[bib53] Osinsky R., Seeger J., Mussel P., Hewig J. (2016). Face-induced expectancies influence neural mechanisms of performance monitoring. Cognit. Affect Behav. Neurosci..

[bib54] Palmer J.A., Kreutz-Delgado K., Makeig S. (2012).

[bib55] Pastötter B., Berchtold F., Bäuml K.-H.T. (2012). Oscillatory correlates of controlled speed-accuracy tradeoff in a response-conflict task. Hum. Brain Mapp..

[bib56] Pastötter B., Dreisbach G., Bäuml K.-H.T. (2013). Dynamic adjustments of cognitive control: oscillatory correlates of the conflict adaptation effect. J. Cognit. Neurosci..

[bib57] Paul K., Vassena E., Severo M.C., Pourtois G. (2020). Dissociable effects of reward magnitude on fronto-medial theta and FRN during performance monitoring. Psychophysiology.

[bib58] Pavone E.F., Tieri G., Rizza G., Tidoni E., Grisoni L., Aglioti S.M. (2016). Embodying others in immersive virtual reality: electro-cortical signatures of monitoring the errors in the actions of an avatar seen from a first-person perspective. J. Neurosci..

[bib59] Philiastides M.G., Biele G., Vavatzanidis N., Kazzer P., Heekeren H.R. (2010). Temporal dynamics of prediction error processing during reward-based decision making. Neuroimage.

[bib60] Purcell B.A., Kiani R. (2016). Neural mechanisms of post-error adjustments of decision policy in parietal cortex. Neuron.

[bib61] Rommerskirchen L., Lange L., Osinsky R. (2021). The reward positivity reflects the integrated value of temporally threefold-layered decision outcomes. Psychophysiology.

[bib62] Ruiz M.H., Strübing F., Jabusch H.-C., Altenmüller E. (2011). Eeg oscillatory patterns are associated with error prediction during music performance and are altered in musician's dystonia. Neuroimage.

[bib63] Smith N.J., Kutas M. (2015). Regression-based estimation of ERP waveforms: I. The rERP framework. Psychophysiology.

[bib64] Spinelli G., Tieri G., Pavone E.F., Aglioti S.M. (2018). Wronger than wrong: graded mapping of the errors of an avatar in the performance monitoring system of the onlooker. Neuroimage.

[bib65] Strack G., Kaufmann C., Kehrer S., Brandt S., Stürmer B. (2013).

[bib66] Töllner T., Wang Y., Makeig S., Müller H.J., Jung T.-P., Gramann K [Klaus] (2017). Two independent frontal midline theta oscillations during conflict detection and adaptation in a simon-type manual reaching task. J. Neurosci..

[bib67] Umemoto A., Lin H., Holroyd C.B. (2023). Electrophysiological measures of conflict and reward processing are associated with decisions to engage in physical effort. Psychophysiology.

[bib68] van de Vijver I., Ridderinkhof K.R., Cohen M. (2011). Frontal oscillatory dynamics predict feedback learning and action adjustment. J. Cognit. Neurosci..

[bib69] van Noordt S.J.R., Desjardins J.A., Gogo C.E.T., Tekok-Kilic A., Segalowitz S.J. (2017). Cognitive control in the eye of the beholder: electrocortical theta and alpha modulation during response preparation in a cued saccade task. Neuroimage.

[bib70] Vocat R., Pourtois G., Vuilleumier P. (2011). Parametric modulation of error-related ERP components by the magnitude of visuo-motor mismatch. Neuropsychologia.

[bib71] Wang C. (2005). Responses of human anterior cingulate cortex microdomains to error detection, conflict monitoring, stimulus-response mapping, familiarity, and orienting. J. Neurosci..

[bib72] Watanabe T., Mima T., Shibata S., Kirimoto H. (2021). Midfrontal theta as moderator between beta oscillations and precision control. Neuroimage.

[bib73] Yeung N., Botvinick M.M., Cohen J.D. (2004). The neural basis of error detection: conflict monitoring and the error-related negativity. Psychol. Rev..

[bib74] Zuure M.B., Hinkley L.B., Tiesinga P.H.E., Nagarajan S.S., Cohen M. (2020). Multiple midfrontal thetas revealed by source separation of simultaneous MEG and EEG. J. Neurosci..

